# Perceived Stress, supportive dyadic coping, and sexual communication in couples

**DOI:** 10.1177/0265407521996446

**Published:** 2021-02-24

**Authors:** Jennifer Yurkiw, Matthew D. Johnson

**Affiliations:** 3156 University of Alberta, Canada

**Keywords:** Coping, couples, intimate relationships, longitudinal, sexual communication, stress

## Abstract

This study investigated associations between perceived stress and sexual communication, considering supportive dyadic coping as a potential mediator and whether being male or female moderated associations. Data from 2,529 couples from Wave 5 of the German Family Panel (pairfam) were used in the analyses. Structural equation modeling results showed higher levels of stress were linked with lower levels of dyadic coping and higher levels of dyadic coping were associated with higher levels of sexual communication. There was no direct association between stress and sexual communication, but there was an indirect relationship between higher levels of perceived stress and less sexual communication via supportive dyadic coping. Sex did not moderate these associations. These results highlight supportive dyadic coping as an important protective factor against the effects of perceived stress on sexual communication and call for further investigation of how couples can maintain a healthy sex life in the face of stress.

Stress influences various aspects of couple sexuality, including reducing sexual satisfaction (Bodenmann et al., 2010) and arousal (Hamilton & Meston, 2013). Impaired sexual relations, in turn, contribute to problems within an intimate relationship (Bodenmann et al., 2010; Impett et al., 2019). Given these associations, the ability of intimate partners to provide emotional and practical support to one another during times of stress, referred to as supportive dyadic coping (Revenson et al., 2005), may serve as a couple-level process that buffers couple sexual communication from the potentially destructive influence of stress. Little research has considered this possibility. This study examines two research questions: (1) How are perceived stress, supportive dyadic coping, and sexual communication associated in couples? and (2) Do the associations among constructs differ for males and females?

## Background

The present study is grounded in a perspective that frames human development as arising from reciprocal influence between person and context (e.g., Lerner et al., 2013). It considers the mutually influential connections between individual-level phenomena and contextual influences, such as one’s intimate partner (Lerner et al., 2013). This perspective prompts consideration of how one’s perceived stress, (an individual-level concern) influences relationship processes in couples, such as supportive dyadic coping, which may then determine couple outcomes, such as sexual communication.

Correspondingly, the stress-coping cascade model (Bodenmann, 2005) posits that when individual coping efforts are insufficient to manage stress, support is sought from the intimate partner. If couples respond to one another’s stress in ways that are constructive and supportive, both partners experience enhanced relationship well-being (Johnson et al., 2016). A relational atmosphere infused with supportive dyadic coping, which includes listening to the partner and communicating understanding of the partner’s perspective regarding their stress, is one that may lead to high quality communication in other parts of the relationship (Cupach & Comstock, 1990), including the difficult area of expressing one’s sexual needs and desires (Theiss & Estlein, 2014).

The research on couple sexual outcomes and stress found, unsurprisingly, higher stress levels are associated with less sexual satisfaction and frequency (Bodenmann et al., 2010). This study focuses on sexual communication, understood as disclosures around sexual preferences (Rancourt et al., 2016), a previously unexamined sexual outcome in relation to stress. Understanding factors that influence sexual communication is important given prior research demonstrating associations between better sexual communication and higher relationship and sexual satisfaction (Cupach & Comstock, 1990; Rancourt et al., 2016). Although critical for healthy sexual relationships, research indicates that many people do not communicate openly with their partners about sexual desires (Theiss & Estlein, 2014).

Supportive dyadic coping may serve an important role linking stress and sexual communication. Sexual communication allows partners to inform one another about their sexual needs and preferences, a process which requires the ability to express oneself openly (Cupach & Comstock, 1990). The unique capacity for supportive dyadic coping to facilitate emotional closeness between couples (Rusu et al., 2020) signifies a possible link to sexual communication insofar as communication is often the vehicle with which couples experience intimacy (Reis & Shaver, 1997). A study from Rusu et al. (2020) found perceived supportive dyadic coping efforts of one’s partner and couple-level dyadic coping predicted higher relationship satisfaction for stressed couples. A daily diary study found that overall dyadic coping did not moderate the association between stress and sexual satisfaction and frequency (Bodenmann et al., 2010). It is possible that couple coping may function as a mediator in the stress to couple sexual communication pathway, as the stress of one partner likely exerts its influence on couple outcomes through the couple’s coping efforts. Additionally, providing support to a partner is a process likely dependent on communication; successful communication around stress may more directly influence communication around sex than satisfaction and frequency.

Based on evidence that dyadic coping is differentially associated with mental health constructs for males and females (Johnson et al., 2017), we also test whether the associations differ for males and females. We include relationship satisfaction and duration as control variables in the analysis given their documented associations with our focal constructs of interest (e.g., Rancourt et al., 2016; Story & Bradbury, 2004).

## Method

### Procedures

This study uses cross-sectional data from 2,529 opposite-sex couples from Wave 5 of the German Family Panel (pairfam) study (Huiniuk et al., 2011). In 2008/2009 (Wave 1) of the study, 3,729 anchor-partner pairs were recruited. The current study uses survey data from anchor participants and their partners in Wave 5 (2012/2013) because this wave was the first to include the focal items under investigation. Data for anchor participants are gathered through computer-assisted personal interviews and self-interviews for sensitive information, including questions about couple sexuality. Partner data are collected through paper and pencil questionnaires. Additional details can be found in the pairfam concept paper (Huinink et al., 2011) and website: http://www.pairfam.de/en/study.html.

### Sample

Given the focus of this study, we filtered the sample to include couples who reported having sexual intercourse in the past (n = 2,259 opposite-sex couples). In Wave 5, anchor participants (51% female) were between 18 and 42 years old (*M* = 33.89, *SD* = 6.95); their partners ranged from 15 to 71 years (*M* = 34.45, SD = 8.24). Most couples (62.3%) were currently married, 24.4% were cohabiting, and 13.3% were dating. Couples had been partnered for 9.99 years (*SD* = 6.40), on average, and approximately one third of anchor participants (36.3%) and 22.6% of partners had a university degree. One third of the sample was childless (32.9%), 21.8% had one child, 31.0% had two children, and 14.1% had three or more children.

### Measures

#### Perceived stress

Three items adapted from the German version of the perceived stress questionnaire (Fliege et al., 2001) assessed the stress of anchor participants. After being provided the prompt “How did you predominantly feel in the last four weeks?” anchors rated themselves from 1 = *not at all* to 5 = *absolutely* on 3 items: “stressed,” “overburdened,” and “under pressure,” Internal consistency among these items was α = .85.

#### Sexual communication

Anchor participants were asked 2 items adapted from Piles et al. (1999) that assessed the quality and openness of sexual communication: “If I want something specific during sexual contact, I say it or show it,” and “Generally speaking, I can express my sexual needs and desires very well.” Responses ranged from 1 = *not at all* to 5 = *absolutely* and mean scores were computed. Internal consistency was α = .85.

#### Supportive dyadic coping

Self-reported supportive dyadic coping from both anchor and partner participants was assessed with 3 items adapted from the Dyadic Coping Questionnaire (Bodenmann, 2008). Participants were prompted to consider “When your partner is stressed out, how often do you react in the following ways?” and rated themselves on the following 3 items: “I let my partner know that I understand him/her,” “I listen to my partner and give him/her the chance to express himself/herself,” and “I support my partner in concrete ways when he/she has a problem.” Responses ranged from 1 = *never* to 5 = *always* and mean scores were computed for anchors and partners. Internal consistency for anchors was α = .77 and α = .76 for partners.

#### Control variables

Relationship duration and relationship satisfaction for anchors and partners were included as control variables. One item from the Relationship Assessment Scale (Hendrick et al., 1998) assessed relationship satisfaction: “All in all, how satisfied are you with your relationship?” Responses ranged from 0 = *very dissatisfied* to 10 = *very satisfied*.

## Analytic plan

Structural equation modeling (SEM) was used to answer the research questions. Perceived stress was modeled as a latent variable with 3 items as indicators and supportive dyadic coping was modeled as a latent variable with anchor and partner self-reports serving as indicators. This modeling approach captured the shared variance between each partner’s support, a technique known as common fate modeling (see Ledermann & Kenny, 2012) that also accounts for non-independence in partner reports, allowing for the analysis of supportive dyadic coping at the couple level (e.g., how much support is provided as a couple). Missing data were low in this sample, ranging from 0.4% (stress) to 6.6% (sexual communication) and were handled with full-information maximum likelihood estimation. Model fit was evaluated with the following commonly used indices: Chi-square test of model fit (χ^2^), comparative fit index (CFI), Tucker-Lewis index (TLI), and root mean square of approximation (RMSEA). A confirmatory factor analysis (CFA) was conducted to evaluate the measurement of the latent constructs, which indicated an acceptable fit to the data: χ^2^ (16) = 142.903, CFI = .972; TLI = .943; RMSEA = .056 (90% CI = .048–.065).

## Results

Bivariate correlations are shown in Table 1. A multiple group SEM was computed to assess whether sex moderated the links between perceived stress, supportive dyadic coping and sexual communication. Equality constraints were placed on corresponding pathways for males and females one at a time and chi-square difference tests assessed whether the constraints significantly worsened model fit. The freely estimated multiple group model fit the data well: χ^2^ (19) = 26.438; CFI = .998; TLI = .997; RMSEA = .018 (90% CI = .000–.032). The application of equality constraints did not worsen fit for the associations between perceived stress and supportive dyadic coping (χ^2^_diff_ (1) = .105, *p =* .746), sexual communication (χ^2^_diff_ (1) = 1.975, *p* = .160), or the link between supportive dyadic coping and sexual communication (χ^2^_diff_ (1) = 2.762, *p =* .096). Sex did not moderate associations among any constructs, so we proceeded to add the control variables and computed a single group model.

**Table 1.** Bivariate correlations and descriptive statistics (n = 2,529 couples).

Variable	*M*	*SD*	Range	1.	2.	3.	4.	5.	6.	7.
1. Anchor Supportive Dyadic Coping	4.13	.55	1–5	—						
2. Partner Supportive Dyadic Coping	3.94	.61	1–5	.27*	—					
3. Sexual Communication	3.61	.89	1–5	.27*	.11*	—				
4. Perceived Stress	3.00	1.05	1–5	−.10*	−.06*	−.09*	—			
5. Relationship Duration (Years)	9.99	6.40	0–20	−.13*	−.14*	−.12*	−.02	—		
6. Relationship Satisfaction (Anchor)	7.86	2.16	0–10	.32*	.20*	.17*	−.03*	−.02	—	
7. Relationship Satisfaction (Partner)	8.14	1.75	0–10	.28*	.40*	.16*	−.10*	−.04*	.33*	—

*Note*. Observed ranges are displayed for each variable.

*p < .05 (two-tailed).

**Figure 1.**
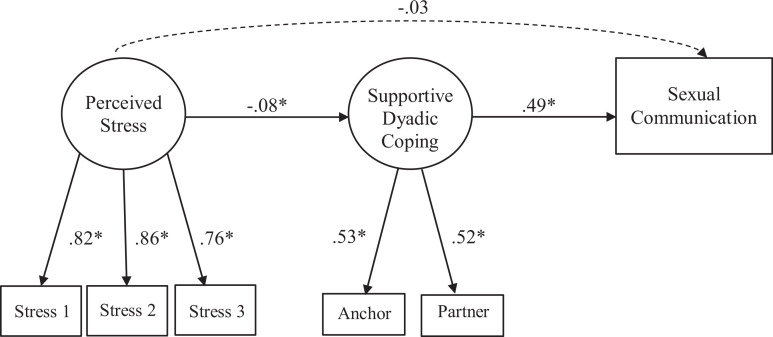
Structural equation model results for associations between stress, supportive dyadic coping, and sexual communication (n = 2,259 couples). *Note.* All variables in the model were also regressed on relationship duration and anchor and partner relationship satisfaction. Model fit indices: χ^2^ (16) = 142.903, CFI = .972; TLI = 0.943; RMSEA = 0.056 (90% CI = .048–.065). *p < .05.

The standardized results for the final model are depicted in Figure 1, which fit the data well. Regarding control variables, relationship duration was not associated with stress but predicted lower sexual communication (β = −.12, *p* < .001) and dyadic coping (β = −.23, *p* < .001). Anchor relationship satisfaction was not associated with sexual communication but was associated with less perceived stress (β = −.12, *p =* .022) and with higher supportive dyadic coping (β = .31, *p <* .001). Partner relationship satisfaction was associated with lower levels of perceived stress (β = −.06, *p* = .010) and sexual communication (β = −.18, *p* < .001), and with higher supportive dyadic coping (β = .53, *p* < .001).

Turning to the key results of interest, higher levels of perceived stress predicted lower levels of supportive dyadic coping and higher levels of supportive dyadic coping were associated with greater sexual communication. The direct association from stress to sexual communication was not significant (*p* = .270). The indirect effect between stress and sexual communication via dyadic coping was tested with 2,000 bootstraps and a 95% confidence interval. The pathway was significant (β = −.04, *p =* .022, 95% CI = −.067, −.015), meaning a 1 standard deviation unit increase in stress is associated with a .04 standard deviation unit decrease in sexual communication via its prior link with dyadic coping.

## Discussion

This study has several notable findings. Higher levels of stress were linked with lower levels of supportive dyadic coping (consistent with prior research; e.g. Bodenmann et al., 2010) and supportive dyadic coping was associated with more sexual communication, a novel finding. This highlights that stress may adversely impact the adaptive coping strategies that couples can use to combat the negative effects of their stress. Supportive dyadic coping is beneficial not only for a couple’s sex life, as supportive dyadic coping has also been linked to higher levels of closeness, commitment, and more secure attachment in couples (Bodenmann et al., 2006; Johnson & Horne, 2016).

Consistent with our theoretical perspectives (Bodenmann, 2005; Lerner et al., 2013), the results support the linked nature of individual and couple well-being. Results showed the direct association between stress and sexual communication was not significant. Rather, stress was associated with lower levels of sexual communication indirectly via its effect on couple coping; stress may impair sexual communication only *through* the couple’s ability to manage stress. The effect size of this pathway was relatively small, and given the exploratory nature of this study, further research exploring these links would be crucial in untangling the potential effectiveness of targeting supportive dyadic coping to mitigate the influence of stress on couple sexuality. Yet, these results do underscore the importance of interventions that teach couples to more effectively support one another during times of stress and also help them implement those strategies at the times when they are needed.

Results also showed the pattern of associations among stress, supportive dyadic coping, and sexual communication were consistent for males and females. These findings contrast prior research that found female’s self-esteem and depressive symptoms were more predictive of supportive dyadic coping than males’s (Johnson et al., 2017), which may signify important disparities in how various facets of mental health, such as stress and depressive symptoms, may differentially impact dyadic coping among the sexes.

Findings from this study must be considered in light of the limitations. First, due to the cross-sectional study design, temporal associations between the variables in the model cannot be established. Although theoretical and empirical evidence suggests that perceived stress would activate couple support (or not; e.g. Bodenmann, 2005) which influences sexual outcomes (Rancourt et al., 2016), it is plausible that these variables operate in a different manner. For example, perceived stress and sexual communication may exhibit bidirectional links with supportive dyadic coping; supportive coping efforts may reduce perceived stress and frequent sexual communication may increase the couple’s willingness to support one another during times of stress. Accordingly, future research should assess links between stress, coping efforts, and sexual communication across time. Next, correlations between anchor and partner supportive dyadic coping were modest (*r* = .27), signifying most of the variation in coping was at the individual level rather than at the level of the dyad. Additional inquiry with more robust measurement to shed light on whether supportive dyadic coping is largely an individual or couple-level pursuit is important. We filtered the sample to include only those who reported having had sexual intercourse in the past, but sexual communication likely plays an important role for those who have never had intercourse but engage in other sexual activities.

While stress may be harmful for relationship functioning (Neff & Karney, 2009), our finding that higher supportive dyadic coping was associated with higher quality sexual communication points to the importance of developing strong shared stress management skills that may ripple out to reinforce positive sexual interactions. In light of evidence that both supportive dyadic coping (Bodenmann et al., 2010) and positive sexual relations (Ein-Dor & Hirschberger, 2012) can relieve stress, it is important to consider the interconnectedness of these factors in stress management.
